# Krüppel-Like Factor 4 Acts as an Oncogene in Colon Cancer Stem Cell-Enriched Spheroid Cells

**DOI:** 10.1371/journal.pone.0056082

**Published:** 2013-02-13

**Authors:** Zhengwei Leng, Kaixiong Tao, Qinghua Xia, Jun Tan, Zhongyi Yue, Jinhuang Chen, Hailin Xi, Jie Li, Hai Zheng

**Affiliations:** 1 Department of General Surgery, Union Hospital, Tongji Medical College, Huazhong University of Science and Technology, Wuhan, People’s Republic of China; 2 Department of Pediatrics, Tongji Hospital, Tongji Medical College, Huazhong University of Science and Technology, Wuhan, People’s Republic of China; The University of Hong Kong, China

## Abstract

Cancer stem cells (CSCs), a rare population in any type of cancers, including colon cancer, are tumorigenic. It has been thought that CSCs are responsible for cancer recurrence, metastasis, and drug resistance. Isolating CSCs in colon cancers is challenging, and thus the molecular mechanism regulating the self-renewing and differentiation of CSCs remains unknown. We cultured DLD-1 cells, one of types of cells derived from colon cancers, in serum-free medium to obtain spheroid cells. These cells possessed the characteristics of CSCs, with the expression of CD133, CD166, Lgr5, and ALDH1, higher capacities of chemo-resistance, migration, invasion, and tumorigenicity *in vitro* and *in vivo* than the adherent DLD-1 cells. Krüppel-like factor 4 (KLF4) is essential factor for maintaining self-renewal of adult and embryonic stem cells. It has been used to induce pluripotent stem cells (iPS) from somatic cells. Since KLF4 is expressed in colon cancer cells, we investigated its role in spheroid cells isolated from DLD-1 cells and found that KLF4 was overexpressed only in spheroid cells and reducing the expression of KLF4 by short-hairpin RNA significantly decreased the capacities of these cells to resist the chemicals, migrate, invade, and generate tumors *in vitro* and *in vivo*. The spheroid cells with reduced KLF4 expression also had decreased expression of CSCs markers and mesenchymal markers. Taken together, culturing DLD-1 cells in serum-free medium enriches CSCs and the expression of KLF4 is essential for the characteristics of CSCs in DLD-1; thus KLF4 can be a potential therapeutic target for treating colon cancer.

## Introduction

Colon cancer is the common cause of cancer-related deaths worldwide [1], [2]. Current therapies, including surgery, radiotherapy, and chemotherapy, were designed to target all tumor cells. The success of these therapies is severely hampered mainly due to the existence of therapy-resistant tumor cells, which are able to develop to entire colon cancer after therapies [3]. It has been thought that these cells are cancer stem cells (CSCs), which have capacity to self-renew and differentiate to all types of cells in the colon cancer [4]. CSCs exist in various types of cancers. They are rare in cancers, but very tumorigenic and also responsible for cancer progression. When transplanted in immunodeficient mice, CSCs show high tumorigenecity. It has also been demonstrated that CSCs are cells responsible for cancer recurrence, metastasis, and drug resistance [5], [6]. Therefore, an effective therapy to treat a cancer requires it to effectively target CSCs in the cancer [7].

CSCs in various types of cancers possess specific surface markers which allow scientists to isolate them. In colon cancer, CSCs are positive for CD133+, CD44+, CD166, Lgr5, ALDH1, and ESA. Colon CSCs also have ability to expel Hoechst 33342 dye [8]–[19]. However, the CSCs are very rare, and in many cases, they cannot be detected. Thus, isolation of CSCs in colon cancers based on their surface markers becomes extremely difficult [20].

CSCs in some cancer cell lines, including colon cancers, can be isolated by culturing cells in serum-free medium to form spheres. In this condition, Spheroid cells, which express “stemness” related genes and possess high capacity to migrate, invade, and generate tumors [20], [21], [22], [23]. In the serum-free medium, many human colon cancer cell lines, including HCT116, HT29, LOVO, SW480 and DLD-1 cell line, can form spheres [22].

The mechanisms by which CSCs from various types of cancers maintain their “stemness” have been extensively studied. The capacity of self-renewal is pivotal for CSCs maintaining and for cancer recurrence [12], [23], [24], [25], [26], [27] KLF4 is essential to maintain the characteristic of CSCs and required for the capacity to migrate and invade in breast cancer [28]. Many groups reported that the KLF4 is expressed and functions as a tumor suppressor in colon cancer [29], [30], [31], [32]. However, as reported, the bulk cells of tumor are unable to generate new cells. While CSCs can regenerate all the cell types in the tumor through their stem cell-like behavior and cause the relapse of cancer [6]. Thus, CSCs and the bulk of cancer cells are extremely heterogeneous. Those studies did not distinguish colon CSCs from the bulk cancer cells.

KLF4 is a zinc finger transcription factor. It has been demonstrated that KLF4 regulates the proliferation, differentiation, apoptosis, and metabolism of thymocyto and colon goblet cell, respectively [33], [34]. In murine, KLF4 is essential for the self-renewal of embryonic stem cells [35]. KLF4 is also used to reprogram mouse fibroblasts to pluripotent stem cells, implying the role of KLF4 on maintaining the characteristics of stem cells [36]. Importantly, the normal stem cells demonstrate common characteristics to cancer stem cells, it will also be important to determine whether similar regulation occurs in stem cells and cancer stem cells. Thus, whether KLF4 is expressed in colon CSCs and its functions of KLF4 in colon cancer need to be addressed.

In this study, we explored the potential roles of KLF4 in CSCs-enriched spheroid cells. We first cultured DLD-1 cells in serum-free medium to get spheroid cells possessing the characteristics of CSCs, and then we asked whether KLF4 were overexpressed in the spheroid cells and whether reduced KLF4 expression in spheroid cells would disturb their characteristics.

## Materials and Methods

### Ethics Statement

This study was carried out in strict accordance with the recommendations in the Guide for the Care and Use of Laboratory Animals of the National Institutes of Health. The protocol was approved by the Committee on the Ethics of Animal Experiments of the University of Huazhong University of Science and Technology (Permit Number: S255). All surgery was performed under a mixture of ketamine and chlorpromazine anesthesia, and all efforts were made to minimize suffering.

### Cell Lines

Human colon cancer cell lines DLD-1, SW480, SW620, LOVO and human colon normal epithelium FHC cell line were commercial sources, and the medium of each cell line was decided according to the American type culture collection (ATCC) or to the published reference. DLD-1, SW620 and SW480 cells were cultured in RPMI1640 (Hyclone) complete medium [37]. Other colon cancer cell lines, HCT116 and HT29 were kindly provided by Dr LIU and were cultured in McCoy’s 5A (Sigma) complete medium [38]. Human colon normal epithelium FHC cells were grown in DMEM/F12 (Hyclone) complete medium. LOVO cells were cultured in DMEM (Hyclone) complete medium [39].

The sphere culture was carried out as described before [22]. Briefly, each line of colon cancer cells were grown at a density of 2×10^6^ cells/ml in serum-free DMEM/F12 medium containing 20 ng/ml epidermal growth factor (EGF, PeproTech), 10 ng/ml basic Fibroblast Growth Factor (bFGF, PeproTech), 5 µg/ml insulin (Sigma), 0.4% bovine serum albumin (Amresco), and 2% B27 (Invitrogen). To passage spheres, we collected them by centrifuging them at 73 g for 5 minutes. Spheres were dissociated to single cells using trypsin digestion. Single spheroid cells were grown as described above. All cells were maintained in a humidified incubator at 37°C and 5% CO2.

### Isolation of CD133+ and CD133− Cells

CD133+ and CD133− cells were isolated from cell culture by magnetic bead sorting using the MACS system (Miltenyi Biotech) or by FACS [12], [40]. For both separations, cells were incubated with the monoclonal CD133/2-PE (Miltenyi Biotech) for 15 min at 4°C. For magnetic separation, cells were selected by MS columns (Miltenyi Biotech), which retained positive cells linked by beads. For FACS separation, CD133/2-PE stained cells were sorted with the BD FACSCanto II Flow Cytometer instrument (BD Bioscience), and the isotype IgG2b (Miltenyi Biotech) was used as the control.

### Lentiviral Infection of Spheroid Cells

Lentiviral vectors bearing KLF4 shRNA (NM004235.3, 1582–1610) or a scrambled non-target shRNA were purchased from Shanghai GeneChem Co., Ltd. The spheroid cells were infected according to the manufacturer’s instructions.

### Real-time PCR

Total mRNAs were extracted from cells and then reversely transcribed to cDNAs with PrimeScript RT Master Mix (Takara, Japan) according to the manufacturer’s instructions. To measure the mRNA levels of genes in each sample, intercalator-based real-time PCR was used. Glyceraldehyde-3-phpsphate-dehydrogenase (GAPDH) was used as an internal control. The real-time PCR was carried out with SYBR Green Master Mix (Takara, Japan) in the StepOnePlus™ Real-time PCR system (Applied Biosystems, USA). PCR amplification of genes was performed under the conditions: denature at 95°C for 30 s, anneal at 60°C for 60 s, and extended at 95°C for 5 s; reactions were carried out for 45 cycles. For each sample, reactions were duplicated and the average mRNA level of each gene was determined using the 2^−ΔΔCt^ method. The primer pairs for measuring the levels of each gene are listed in the Table S1.

### Immunofluorescence Staining

Expressions of CD133, KLF4, E-cadherin, and Vimentin in monolayer cells and spheres were also analyzed with immunofluorescence technique. To carry out immunofluorescence analysis, cells were grown in appropriate medium onto glass-coverslips for 16 hours before fixed with 2% paraformaldehyde at 37°C for 15 min. Fixed cells were then permeablized with 0.1% Triton X-100 at room temperature for 15 min followed by incubating at 4°C for overnight with the following primary antibodies: CD133/2 (Miltenyi Biotec), E-cadherine (Santa Cruz Biotec), Vimentin (Cell Signaling Technology), and KLF4 (Santa Cruz Biotec), respectively. On next day, cells were then incubated with PE- or FITC-conjugated secondary antibody (Boster) at room temperature for 1 hour. These slides were then mounted with Prolong Gold with 4′, 6-diamidino-2-phenylindole (DAPI, Invitrogen). Fluorescent images were taken using Olympus IX71 epifluorescence (Olympus, Japan).

### Western Blot Analysis

Protein extracts were separated by electrophoresis on various concentrations of SDS-polyacrylamide gels depending on the expected sizes of measured proteins and transferred to PVDF membranes (Millipore, USA). After blocked with 5% nonfat milk and 0.1% Tween 20 in TBS for 1 h, the membrane was then incubated at 4°C for overnight with the primary antibodies followed by incubating with Goat anti-rabbit secondary antibodies. Primary antibodies used in this analysis are: rabbit anti-E-cadherin, rabbit anti-Vimentin, rabbit anti-ZO-1, and rabbit anti-Snail (Cell Signaling Technology); rabbit anti-KLF4 and rabbit anti-Lgr5 (Santa Cruz Biotec).

### Chemotherapy Sensitivity and Resistance Assay

The sensitivity of colon cancer cells to 5-FU was analyzed using CCK-8 assay kit (DOJINDO, molecular technolonies, Inc, Japan) according to manufacturer’s instruction. Briefly, 2,000 cells were seeded into each well of 96-well plates containing growth medium supplemented with various concentrations of 5-FU (Sigma) and incubated at 37°C and 5% CO_2_ in a humidified condition for 48 hours. The absorption of each well was read at 450 nm in a plate reader. The survival rate of cells was calculated as follows: absorption of experiment/absorption of control × 100%.

### Annexin-V APC/PI Double Labelling

Cell were collected, washed with phosphate-buffered saline (PBS) and stained with Annexin-V APC (Keygen, China) and PI (Sigma) both for 30 min at room temperature in the dark according to the manufacturer’s instructions. All stained cells were then examined by using BD FACSCanto II Flow Cytometer instrument (BD Bioscience).

### Soft Agar Assay

Soft agar assay was performed for spheroid cells as previously described [41]. Briefly, each well of a 6-well plate was loaded with 2 mL 0.5% agar (w/v) in RPMI1640 supplemented with 10% FBS as base. After polymerization of the base agar, 1×10^4^ cells mixed in 2 mL 0.375% agar (w/v) in RPMI1640 supplemented with 10% FBS were added. Cultures were maintained in a humidified incubator at 37°C and 5% CO_2_ for 2 weeks before colonies were counted and photographed.

### Colony Formation Assay

Colony formation assay was performed for adherent DLD-1 cells as previously described [27]. Briefly, single cells (2,000 cells per well) were plated in 6-well plates and cultured for 2 weeks. After stained with violet, cells were photographed and analyzed for their proliferation and colony formation efficiency. All the experiments were performed at least three times.

### Cell Migration and Invasion Assay

Migration and invasion assay was carried out as described before [42]. Briefly, 10^6^ cells were plated onto cell culture inserts with 8 µm microporous filters (BD Bioscience) coated with (invasion) or without (migration) matrigel (Sigma) and incubated for 20 h, respectively. The migrated or invaded cells were stained with 0.1% crystal violet and counted in five random fields. Experiments were performed in triplicate.

### *In vivo* Tumorigenesis

Cells were obtained by trypsinized harvested spheres. Various amounts of cells (1×10^4^, 5×10^4^, 1×10^5^, 5×10^5^, or 1×10^6^ cells) in 200 µl PBS were subcutaneously transplanted into 4- to 6-week-old athymic female, Balb/c nu/nu mice (Beijing HFK Bioscience). The tumor size was measured every 4 days using a caliper. The volume of each tumor was determined using the formula: length×width^2^×0.5. All animal work had been conducted according to the guidline of the Ethics Commission of Huazhong University of Science and Technology (S255). Mice were housed in a specific pathogen-free, environmentally controlled facility. Mice were sacrificed with pentobarbital sodium intraperitoneal injection and the grafts were removed when tumors reached a length of 2.0 cm, or 60 days after injection, whenever was first [41]. Harvested tumors were prepared for histopathologic analysis.

### Histopathologic Analysis

Tumors were harvested and fixed in 4% formalin for 24 hours before embedded in paraffins. Sections (2.5 µm) were obtained and stained with H&E. Images were taken with Olympus IX71 (Olympus, Japan).

### Statistical Analysis

Each experiment was performed at least three independent trials. The results were expressed as the mean ± SD. Statistical analyses were performed using a Student’s *t* –test, where *P*<0.05 was considered statistically significant.

## Results

### Generation of Spheroid Cells from Colon Cancer Cell Lines

**Figure 1:**
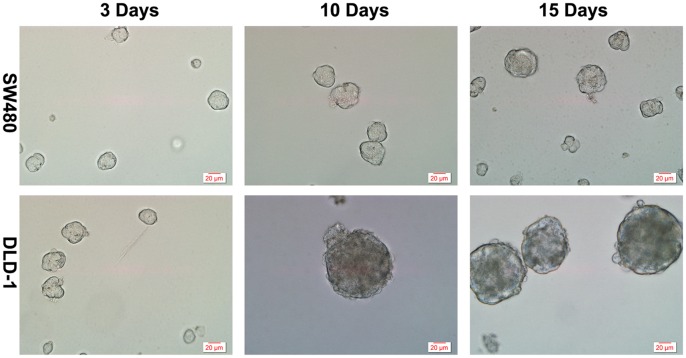
Spheres generated and passaged *in vitro*. HT29, HCT116, SW620, LoVo, and DLD-1 cells could form large round, unattached floating colonsphere of 50–100 µm when cultured in SFM (as shown in DLD-1 group). While SW480 could not.

Previous studies showed HCT116 and HT29 colon cancer cells form spheres when cultured in serum-free medium and the spheroid cells possesses the characteristics of CSCs [22], [43], [44]. We asked whether HCT116, HT29, SW620, SW480, LOVO, and DLD-1 cells could form spheres when cultured in serum-free medium, respectively. Cells from each cell line were cultured at a density of 2×10^6^/ml in serum-free medium. On day 3, we could see sphere formation from HCT116, HT29, SW620, LOVO, and DLD-1, but not from SW480 (Figure 1 and data not shown). The spheres increased in size along the time. Furthermore, the spheroid cells in the spheres could be passaged for more than 25 times without loss of any characteristics tested below, indicating that they might possess capacity to self-renew. Because DLD-1 cells form spheres more easily than other colon cancer lines tested above, we used only focused on DLD-1 cells in following experiments. For our convenient description, we designated the spheroid cells from DLD-1 as DLD-S.

### Characterization of Spheroid Cells

**Figure 2:**
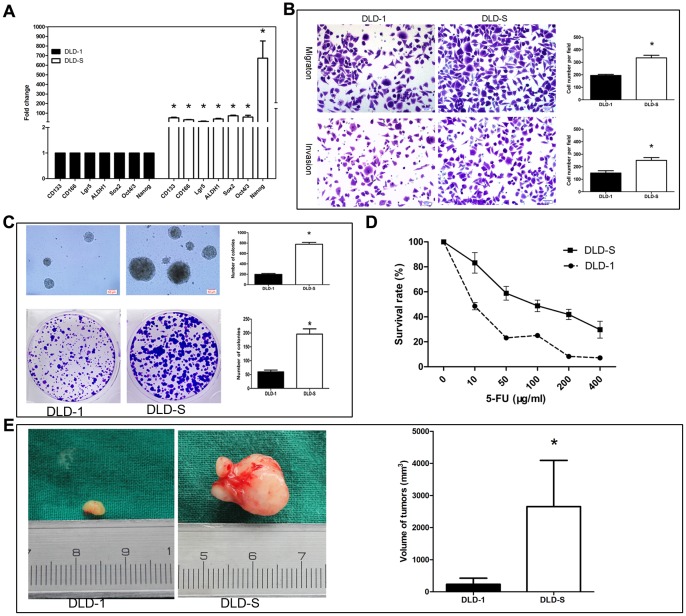
Characterization of spheroid cells. (A) The expression of CD133, CD166, Lgr5, ALDH1 and stem cell core genes Oct4/3, Sox2 and Nanog in spheroid cells were up-regulated as compared to DLD-1 adherent cells. (B) More spheroid cells were obviously observed as compared to their parental DLD-1 cells, analyzed using transwell migration and invasion assay, respectively. (C) As assessed by soft agar assay and colony formation assay, the spheroid cells exhibit higher capacity of colony formation. (D) As assessed by the CCK8 assay, survival rate of spheroid cells is increased as compared to adherent cells in various concentrations of 5-FU. (E) As assessed by mouse xenograft model, the spheroid cells had higher tumorigenic ability than their parent DLD-1 cells. *P<0.05.

We first asked whether DLD-S expressed the surface markers of CSCs of colon cancer and the stem cell core genes. We found that the expression of the stem cell core genes such as Oct4/3, Sox2 and Nanog and the marker genes of colon CSCs, such as CD133, CD166, Lgr5, and ALDH1 were increased in DLD-S cells when compared with their expression levels in DLD-1 (Figure 2A), suggesting that spheroid cells might have high percentage of CSCs.

We then investigated the malignant profile of DLD-S cells, including their ability to migrate and invade, to form colonies, to respond to 5-FU, and to generate tumors in immunodeficiency mice. Using transwell migration/invasion assay, we found that DLD-S cells had significantly higher capacity to migrate and invade Matrigel-coated inserts than their parent cells, DLD-1, did (Figure 2B).

Using soft agar assay and colony formation assay, we found that DLD-S had significant higher colony formation capacity than their parent cells, DLD-1 (Figure 2C).

To test the sensibility of DLD-S to chemotherapy, we treated cells with 5-FU at various concentrations. The survival rates of DLD-S and DLD-1 in growth medium with same concentration of 5-FU were compared, and we found that DLD-S had significantly higher survival rate in tested concentrations of 5-FU than their parent cells, DLD-1 cells did, indicating that DLD-S were more resistant to chemotherapy (Figure 2D).

Finally, we investigated the tumorigenic ability of DLD-S cells *in vivo*. Various amount of DLD-S or DLD-1 (1×10^4^, 5×10^4^, 1×10^5^, 5×10^5^, or 1×10^6^ cells) were subcutaneously injected into Balb/c nu/nu mice and mice with tumor formation were counted at 60 days after cell transplantation. We found that DLD-S cells had higher tumorigenic ability than their parent cells, DLD-1 (Table S2). A mouse transplanted with 1×10^5^DLD-S or DLD-1 cells at 60 days was shown in Figure 2E.

### Expression of KLF4 in Colon Cancer Cells and CSCs-enriched Populations

**Figure 3:**
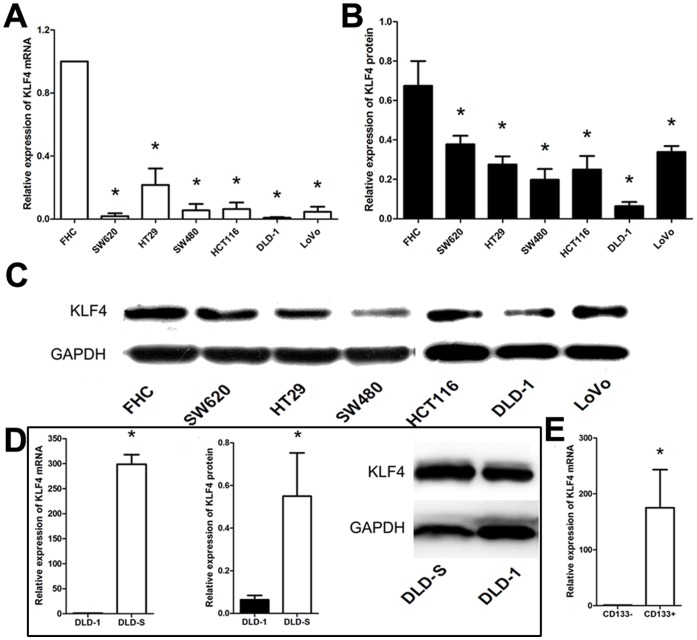
Expression of KLF4 in colon cancer cells and CSCs-enriched populations. (A) As assessed by Real-time PCR, the expression of KLF4 in several colon cancer cell lines was significantly lower than the normal colon epithelial cell line. (B and C) As assessed by Western-blot, the expression of KLF4 in several colon cancer cell lines was significantly lower than the normal colon epithelial cell line. (D) The expression of KLF4 was significantly higher in spheroid cells than in DLD-1, HT29, HCT116 adherent cells as assessed by Real-time PCR and Western-blot, respectively (as shown in DLD-1 group). (E) As assessed by Real-time PCR, the expression of KLF4 was significantly higher in CD133+ cells than in CD133-cells from DLD-1. *P<0.05.

We first tried to confirm that the expressions of KLF4 in various colon cancer cell lines were lower than the normal colon epithelial cell lines as previously reported [45]. The expression levels of KLF4 in SW620, HT29, SW480, HCT116, DLD-1, and LOVO human colon cancer cell lines as well as FHC, a normal colon epithelial cell line, were analyzed using real-time PCR and Western blotting analysis. As expected, the KLF4 was expressed in all tested cell lines and the mRNA levels (Figure 3A) and the protein levels (Figure 3B and 3C) in these cancer cell lines were significantly lower than the FHC cell line.

**Figure 4:**
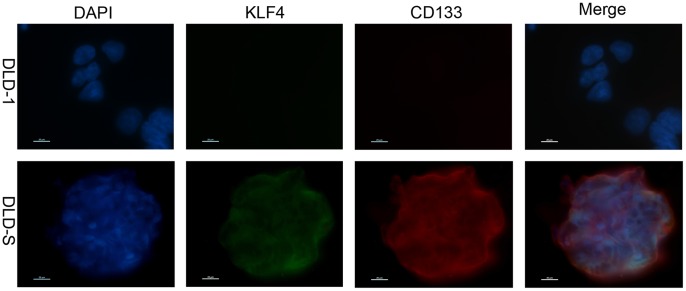
The co-expression of KLF4 and CD133 in DLD-1 and DLD-S cells. Immunofluorescence analysis confirming the presence of CD133 and KLF4 on DLD-1 and DLD-S cells, nucleus is stained with DAPI.

We then compared the expression levels of KLF4 in spheroid cells and adherent cells of DLD-1, HT29, and HCT116 cells, and found that the mRNA and protein levels of KLF4 were significantly higher in spheroid cells than in adherent cells (Figure 3D and date not shown). We further sorted CD133+ and CD133− cells from the DLD-1 cells by MACS separation technology and measured the mRNA levels in both subgroups of DLD-1 cells using real-time PCR. We observed that the mRNA level of KLF4 was significantly higher in CD133+ cells than in CD133− cells (Figure 3E). Expression of CD133 and KLF4 in DLD-S cells was further confirmed by immunofluorescent staining (Figure 4). Taken together, these results suggest that KLF4 is most likely expressed in CD133+ subpopulation and DLD-1-derived spheroid cells, which enriched CSCs, challenging the previous conclusion of that KLF4 functions as a tumor suppressor in colon cancer progression.

### Knockdown of the Expression of KLF4 in Spheroid Cells Altered their Stemness

**Figure 5:**
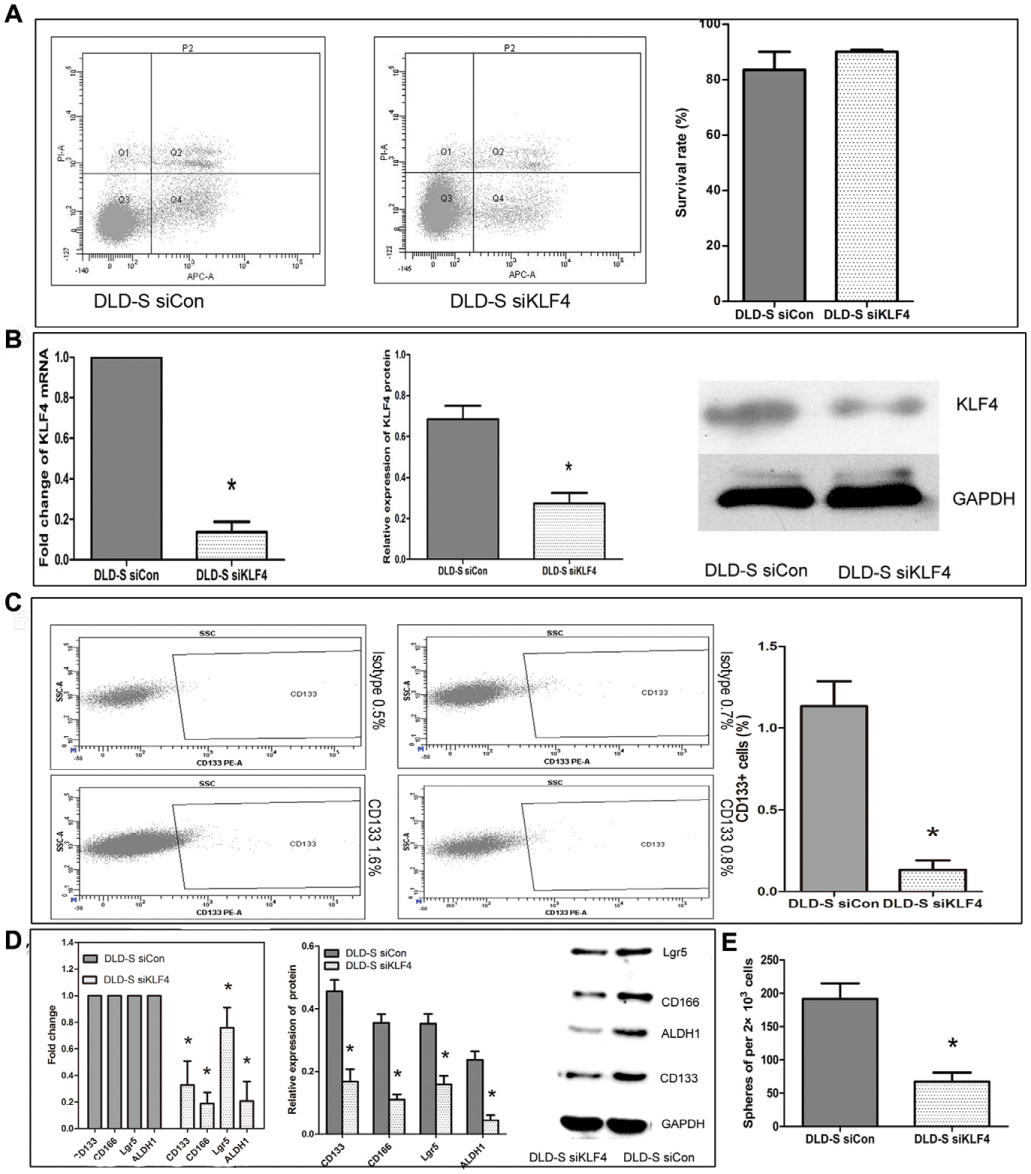
Knockdown of the expression of KLF4 in spheroid cells altered their stemness. (A) The survival rate was similar among both DLD-S siCon and DLD-S siKLF4 cells as assessed by Annexin-V APC/PI Double Labelling assay. (B) The expression of KLF4 was efficiently knocking down in DLD-S cells. (C) Cytometric analyses of CD133+ cells. The CD133+ fraction was significantly decreased after knocking down KLF4 in DLD-S cells. (D) The expression of CD133, CD166, Lgr5, and ALDH1 was significantly lower in DLD-S siKLF4 than in DLD-S siCon. (E) Cultured in SFM, DLD-S siKLF4 cells formed small spheres in a significantly lower frequency than DLD-S siCon cells did. *P<0.05.

We then asked whether KLF4 is essential for the self-renewal and/or the malignant profile of spheroid cells from DLD-1. For this purpose, we generated DLD-S siKLF4 by infecting DLD-S cells with KLF4-shRNA lentiviral vector and the DLD-S siCon by infecting DLD-S cells with the non-target shRNA lentiviral vector. We first examined the apoptosis of DLD-S siCon and DLD-S siKLF4 cells to exclude the possibility that the decreased capability of chemo-resistance, migration, invasion and tumorigenicity of siKLF4 DLD-S cells was due to increased apoptosis by knocking down KLF4 expression and found that the survival rate was similar among both DLD-S siCon and DLD-S siKLF4 cells (Figure 5A). Then, we studied the mRNA and protein levels of KLF4 in these viral infected DLD-S cells and found that both mRNA and protein levels of KLF4 were significantly lower in DLD-S siKLF4 cells than in DLD-S siCon cells (Figure 5B), suggesting that KLF4 gene expression was successfully knocked down.

We then asked whether knockdown of KLF4 expression in DLD-S altered their CSC-marker gene expression by using FACS analysis, real-time PCR, and Western blotting analysis. We found that the number of CD133+ cells is significantly lower in DLD-S siKLF4 cells than in DLD-S siCon cells (Figure 5C) and the expression of all CSC-marker genes, including CD133, CD166, Lgr5, and ALDH1, significantly lower in DLD-S siKLF4 than in DLD-S siCon (Figure 5D). Finally, we asked whether knockdown of KLF4 affected the self-renewal of DLD-S. When cultured in serum-free medium, DLD-S siKLF4 cells formed small spheres in a significantly lower frequency than DLD-S siCon cells did (Figure 5E), suggesting that KLF4 is essential for the self-renewal of DLD-S cells.

### Knockdown of the Expression of KLF4 Altered the Malignant Profile of Spheroid Cells of DLD-1 Cells

**Figure 6:**
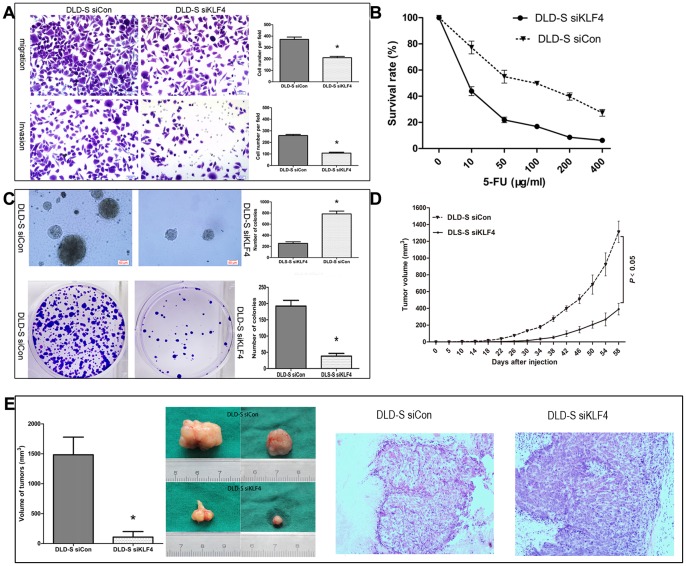
Knockdown of the expression of KLF4 altered the malignant profile of spheroid cells. (A) DLD-S siKLF4 migrated and invaded significantly slower than the DLD-S siCon cells, assessed by transwell assay. (B) As assessed by the CCK8 assay, DLD-S siKLF4 had significant lower survival rates than the DLD-S siCon cells in various concentrations of 5-FU. (C) DLD-S siKLF4 cells formed significant lower number of colonies and smaller colonies than the DLD-S siCon. (D and E) DLD-S siCon cells formed tumors earlier and significantly larger than in mice transplanted with DLD-S siKLF4 cells. There was no significant histological difference between DLD-S siCon and DLD-S siKLF4 groups. Original magnifications ×200. *P<0.05.

We further investigated whether knockdown of KLF4 expression in DLD-S would alter their malignant profile by comparing the ability of DLD-S siKLF4 and DLD-S siCon to migrate and invade, to form colonies, to respond to 5-FU, and to generate tumors in immunodeficiency mice. Firstly, using transwell assay, we found that the DLD-S siKLF4 migrated and invaded significantly slower than the DLD-S siCon cells (Figure 6A). Secondly, we tested the survival rates of the DLD-S siKLF4 cells in growth medium with 5-FU and found that the DLD-S siKLF4 had significant lower survival rates than the DLD-S siCon cells (Figure 6B). Thirdly, using soft agar assay and colony formation assay, we found that DLD-S siKLF4 cells formed significant lower number of colonies and smaller colonies than the DLD-S siCon (Figure 6C). Finally, we used the mouse xenograft model to ask whether KLF4 is required for *in vivo* tumorigenesis of CSC-enriched DLD-S cells. One million DLD-S siKLF4 or the siCon cells were subcutaneously injected into each Balb/c nu/nu mouse, respectively. We found that tumors were formed in mice transplanted with DLD-S siCon cells earlier and significantly larger than in mice transplanted with DLD-S siKLF4 cells (Figure 6D). For example, at day 56 post cell injection, tumors in mice receiving DLD-S siCon grew to an average of 1256.52 mm^3^ in volume, while tumors in mice receiving DLD-S siKLF4 grew only to average of 374.11 mm^3^. Histology of xenograft tumors was examined by HE staining. There was no significant histological difference between DLD-S siCon and DLD-S siKLF4 groups (Figure 6E). Taken together, these results suggested that knockdown of KLF4 expression in DLD-S cells crippled the capacities of these cells to migrate, invade, resist 5-FU, and generate tumors.

### Knocking Down KLF4 Expression Suppresses Epithelial–mesenchymal Transition in Spheroid Cells

**Figure 7:**
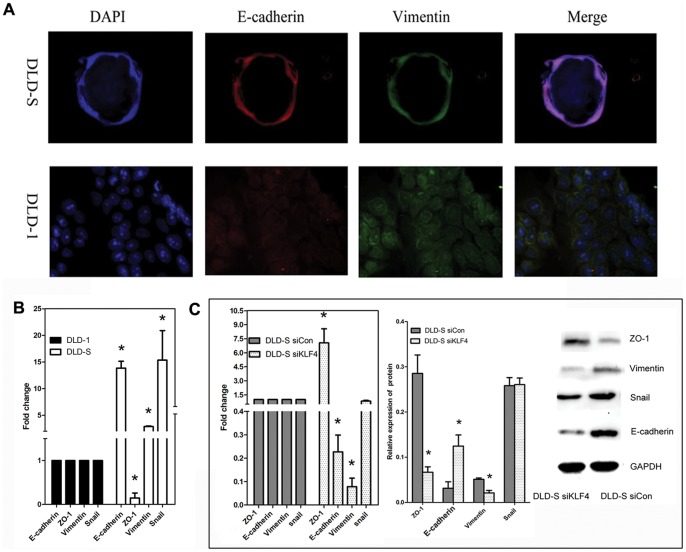
Knocking down KLF4 expression suppresses epithelial–mesenchymal transition in spheroid cells. (A) Immunofluorescence analysis on DLD-S siCon and DLD-S siKLF4 cells for E-cadherin and Vimentin, nucleus is stained with DAPI. (B) As assessed by Real-time PCR, DLD-S cells had significant higher mRNA levels of E-cadherin, Vimentin and Snail. The expression of ZO-1 was decreased. (C) DLD-S siKLF4 cells had significant higher protein levels of ZO-1, but significant lower protein levels of E-cadherin and Vimentin than DLD-S siCon. The expression of snail was similar among them. *P<0.05.

Epithelial-mesenchymal transition (EMT) process is closely related with the metastatic feature of cancer cells [46], [47]. Cancer cells engaging EMT process express mesenchymal genes, such as Vimentin, snail, and slug, while the expressions of epithelial marker genes, such as E-cadherin and ZO-1 are decreased. These cells also have similar malignant profile as CSCs or cancer-initiating cells do. We first demonstrated that, unlike DLD-1 cells, DLD-S expressed a typical epithelial marker, E-cadherin and a typical mesenchymal marker, Vimentin by immunofluorescence and Real-time PCR analysis (Figure 7A and 7B), suggesting that DLD-S did possess the features of cells that go through EMT. We then compared the expression of epithelial and mesenchymal markers among DLD-S siKLF4 and DLD-S siCon cells and found that DLD-S siKLF4 cells had significant higher protein levels of ZO-1, but significant lower protein levels of E-cadherin and Vimentin than DLD-S siCon (Figure 7C). Interestingly, the expression of snail was similar among both DLD-S siKLF4 and DLD-S siCon. These results suggest that KLF4 is required for promoting EMT process in colon spheroid cells.

## Discussion

In this study, we were able to enrich CSCs from several colon cancer cell lines by culturing them in serum-free medium. The spheres formed in the serum-free medium contained spheroid cells, which possessed characteristics of colon CSCs: first, these cells had high expression of marker genes of colon CSCs and stem cells (Figure 2A); second, these cells were more malignant features than their parental DLD-1 cells (Figure 2B, 2C, and 2D); finally, these cells showed increased tumorigenicity when subcutaneously transplanted into immunodeficient mice (Figure 2E). Others also used serum-free medium system to get CSCs-enriched spheres from HCT116, HT29 colon cancer cell lines [22], [43], [44]. It is worth pointing out that this CSCs-enriching method is not suitable for all colon cancer cell lines. For example, in our study, we were not able to enrich spheres from SW480 using this method.

We also concluded that KLF4 was required for the characteristics of colon CSCs. First, KLF4 was only highly expressed in CSC-enriched spheroid cells of DLD-1, HCT116, HT29 cells and CD133+ cells isolated from DLD-1 cells (Figure 3D, 3E, and date not shown). Second, knocking down the KLF4 expression in DLD-S cells reduced the number of CD133+ cells and reduced the expression levels of colon cancer stem cell marker genes (Figure 5C and 5D ) and those cells could not effectively form spheres in serum-free medium (Figure 5E). Third, DLD-S cells with decreased KLF4 expression showed crippled capacity to migrate, invade, resist to 5-FU, and generate tumors (Figure 6A, 6B, 6C, 6D, and 6E). Finally, knocking down KLF4 expression in DLD-S cells inhibited epithelial–mesenchymal transition of these cells (Figure 7C). Moreover, KLF4 was reported to be related to cell apoptosis and survival. We compared the apoptosis of DLD-S siCon and DLD-S siKLF4 cells to exclude the possibility that the decreased malignant profile of DLD-S siKLF4 cells was due to increased apoptosis by knocking down KLF4 expression (Figure 5A). Thus, from our study, KLF4 most likely expresses in colon CSCs and acts as an oncogene for the development of colon cancer.

KLF4 was reported to be significantly lower than normal colonic tissues and FHC [45]. Reconstitution of KLF4 in the colon cancer cell line RKO reduces colony formation, cell migration, and invasion [29]. Up-regulation of KLF4 in HT29 cells induced growth inhibition and cell death [48]. Thus, KLF4 has been thought to be a suppressor in the development of colon cancer. However, Colon cancer cells are extremely heterogeneous and CSCs are very rare. Those studies did not distinguish colon CSCs from the bulk cancer cells and the mechanisms of KLF4 in colon CSCs may not be fully investigated.

As a zinc finger transcription factor, KLF4 plays a role on maintaining the self-renewal of murine embryonic stem cells [35]. Moreover, KLF4 is also used to reprogram mouse fibroblasts to pluripotent stem cells, implying the role of KLF4 on maintaining the characteristics of stem cells [36]. In this study, we reported that spheroid cells exhibit biological similarities with CSCs. Moreover, we found that KLF4 is dramatically overexpressed in CSCs enriched populations - spheroid cells and CD133+ subpopulations. Although we have not performed methods to determine the tumor-initiating capacities of CD133+ cells *in vivo*, the importance of CD133 in CSCs has widely been confirmed [49]–[55] As KLF4 plays a key role in the induction and maintenance of stem cells. Thus, an interesting issue is whether KLF4 acts still as a cancer suppressor in colon CSCs or as a critical factor involved in colon CSCs self-renewal. Thus, the biology of KLF4 in colon CSCs needs further investigation.

It was reported that KLF4 is required for maintenance of breast cancer stem cells [28], [56]. To date, no study has confirmed whether KLF4 is an important regulator of colon cancer stem cell apoptosis and renewal, and whether it plays the vital role in colon CSCs. Our findings here also suggest that although KLF4 was reported as a tumor suppressor, it functions in colon CSCs-enriched spheroid cells for metastatic progression of colon cancer. As we mentioned before, KLF4 exerted an anti-apoptotic function in many cell lines. To exclude the possibility that the reduction of CSCs-enriched population may be a result of the increased apoptosis mediated by KLF4 knockdown, we assessed the survival rate of DLD-S siCon and DLD-S siKLF4 cells by Annexin-V APC/PI Double Labelling assay, and found that no difference among them.

EMT is implicated in physiological processes, such as embryonic development and wound healing and in pathological processes, such as the progression of early-stage non-invasive tumors to invasive malignancies [46]. The process of EMT involves a disassembly of cell-cell junctions, actin cytoskeleton reorganization and increased cell motility, as characterized by decreased of epithelial genes such as E-cadherine and ZO-1 while acquiring mesenchymal molecular such as vimentin, alpha-smooth muscle actin (α-SMA), snail, slug and fibronectin [57]. Importantly, these changes in gene expression are correlated with an increasingly invasive and aggressive tumor cell phenotype that is associated with a poorer patient prognosis [47], [58]. Silencing of vimentin or re-expression of E-cadherin in invasive cells also decreases their invasive phenotype; emphasizing that these genes play a major role in controlling the metastatic behavior of tumor cells. Likewise, the master regulators of EMT have been shown to be associated with increased malignancy and to regulate carcinoma cell invasion and metastasis [59].

It has been reported that forced expression of KLF4 in breast tumor cell line and gastric cancer was sufficient to restore E-cadherine expression and inhibit EMT as well as suppressed migration and invasion [56], [60]. In our study, overexpression of KLF4 in spheroid cells led to a significant induction of E-cadherin, Snail as well as vimentin and deduction of ZO-1, the key mediators of EMT (Figure 7B). Furthermore, knocking down KLF4 expression in DLD-S cells decreased their protein levels of E-cadherin, Vimentin, and increased expressed of ZO-1, in a same pattern from previously reported pattern in regulating E-cadherin while in a different pattern in regulating EMT, migration and invasion. Although several studies have demonstrated that E-cadherin downregulation is a major event during EMT and tumor progression, our study indicates that decreased of E-cadherin may not be the sole contributor to EMT as mentioned by another group [61]. The induction of specific mesenchymal markers in addition to increased or decreased of E-cadherin may be required for full invasiveness during cancer progression. In our current studies, although KLF4 induced expression of E-cadherin, consistent with the induced expression of vimentin and decreased expression of ZO-1 as well as *in vitro* findings, it appears that KLF4 induced EMT in colon CSCs-enriched population.

In summary, we successfully used serum-free culture system to enrich colon CSCs in most colon cell lines. Using these CSC-enriched spheroid cells, we conclude that KLF4, which was previously thought to be a tumor suppressor, functions as an oncogene for the development of colon cancer. Therefore, KLF4 may be a potential target when we develop strategies to treat colon cancer. Our study suggests that CSCs enriched using serum-free culture system can be used to elucidate the molecular mechanisms by which colon cancer develops.

## Supporting Information

Table S1 **Primer names and sequences.**
(DOC)

Table S2 **Tumor-forming efficiency of DLD-1 and DLD-S cells.**
(DOC)
