# Supplementary material for: Krüppel-Like Factor 4 Acts as an Oncogene in Colon Cancer Stem Cell-Enriched Spheroid Cells
Source: PLoS One. 2013 Feb 13;8(2):e56082. doi: 10.1371/journal.pone.0056082 (PMC3572033; doi:10.1371/journal.pone.0056082)
Supplement: Table S1 — Primer names and sequences. (DOC) [file pone.0056082.s001.doc]

**Table S**1: Primer names and sequences.

| **Primer Name** | **Primer Sequence (5’ to 3’)** |
| --- | --- |
| **GAPDH-F** | GGGGAGCCAAAAGGGTCATCATCT |
| **GAPDH-R** | GACGCCTGCTTCACCACCTTCTTG |
| **KLF4-F** | CGAACCCACACAGGTGAGAA |
| **KLF4-R** | TACGGTAGTGCCTGGTCAGTTC |
| **E-cadherin-F** | GCCCTGCCAATCCCGATGAAA |
| **E-cadherin-R** | GGGGTCAGTATCAGCCGCT |
| **ZO-1-F** | TGAGGCAGCTCACATAATGC |
| **ZO-1-R** | GGGAGTTGGGGTTCATAGGT |
| **Vimentin-F** | GCTTCAGAGAGAGGAAGCCGAAAA |
| **Vimentin-R** | CCGTGAGGTCAGGCTTGGAAA |
| **Snail-F** | CCAGACCCACTCAGATGTCAAGAA |
| **Snail-R** | GGCAGAGGACACAGAACCAGAAAA |
| **CD133-F** | ACCGACTGAGACCCAACATC |
| **CD133-R** | GACCGCAGGCTAGTTTTCAC |
| **CD166-F** | CGCAATGCAACAGGAGACTA |
| **CD166-R** | CCACAGTTGCATTCCTGCTA |
| **Lgr5-F** | CTCTTCCTCAAACCGTCTGC |
| **Lgr5-R** | GATCGGAGGCTAAGCAACTG |
| **ALDH1-F** | TGTTAGCTGATGCCGACTTG |
| **ALDH1-R** | CTTCTTAGCCCGCTCAACAC |
| **Oct4/3-F** | CTTGCTGCAGAAGTGGGTGGAGGAA |
| **Oct4/3-R** | CTGCAGTGTGGGTTTCGGGCA |
| **Sox2-F** | CAAGATGCACAACTCGGAGA |
| **Sox2-R** | CATGAGCGTCTTGGTTTTCC |
| **Nanog-F** | CAGAAGGCCTCAGCACCTACCTACCCCAGCC |
| **Nanog-R** | TCTCTGCAGTCCTGCATGCAGTTCCAGCCAAA |
