# Supplementary material for: Krüppel-Like Factor 4 Acts as an Oncogene in Colon Cancer Stem Cell-Enriched Spheroid Cells
Source: PLoS One. 2013 Feb 13;8(2):e56082. doi: 10.1371/journal.pone.0056082 (PMC3572033; doi:10.1371/journal.pone.0056082)
Supplement: Table S2 — Tumor-forming efficiency of DLD-1 and DLD-S cells. (DOC) [file pone.0056082.s002.doc]

**Table S2**

**Tumor-forming efficiency of DLD-1 and DLD-S cells**

| No. of cells injected | DLD-1 tumors formed | DLD-S tumors formed |
| --- | --- | --- |
| 10,000 | 0/7 | 0/8 |
| 50,000 | 0/8 | 2/8 |
| 100,000 | 2/8 | 6/8 |
| 500,000 | 3/8 | 8/8 |
| 1,000,000 | 5/8 | 10/10 |
